# Antibody‐Targeted Artificial T Cell and Natural Killer Cell Derived Vesicles for Cancer Immunotherapy

**DOI:** 10.1002/jev2.70231

**Published:** 2026-01-30

**Authors:** Brijesh Parlekar, David W. Livingstone, Ashley R. Sutherland, Andrés X. Medina, Wendy Bernhard, John DeCoteau, Tays Hernández, Clarence Ronald Geyer

**Affiliations:** ^1^ Department of Health Sciences University of Saskatchewan Saskatoon Saskatchewan Canada; ^2^ Department of Biochemistry, Microbiology and Immunology University of Saskatchewan Saskatoon Saskatchewan Canada; ^3^ Department of Pathology and Laboratory Medicine University of Saskatchewan Saskatoon Saskatchewan Canada; ^4^ Center for Molecular Immunology Havana Cuba

**Keywords:** T cell, NK92 cell, artificial cell‐derived vesicles, click‐chemistry, antibody conjugation, targeted immunotherapy

## Abstract

T cell and NK92 cell‐derived extracellular vesicles or artificial cell‐derived vesicles (ACDVs) exhibit anti‐cancer effects through inherited cytotoxic proteins like perforin and granzyme or transmembrane receptors like FasL and TRAIL. The anti‐cancer potential of artificial T and NK vesicles has been improved by attaching targeting moieties to their surface using genetic engineering or covalent surface modifications. However, the genetic engineering of immune cells from which the vesicles are derived is laborious, expensive, and inefficient, and naturally derived exosomes are released in low quantities. Here, we compared the properties of tumour‐targeted and untargeted ACDVs from activated‐T cells and NK92 cells.

We examined whether the cancer cell‐killing capacity of ACDVs derived from activated‐T and NK92 cells could be targeted to cancer cells by conjugating tumour‐targeting antibodies to their surface. We targeted T and NK92 ACDVs to cancer cells possessing the xenoantigen, N‐glycolyl neuraminic acid GM3 ganglioside, using the 14f7hT antibody or the tumour antigen, epidermal growth factor receptor, using the nimotuzumab antibody. Antibody targeting improved the cell interaction, internalization, and cytotoxicity of T and NK92 ACDVs. Interestingly, the T‐ACDVs retained perforin, granzyme, FasL and TRAIL, whereas NK92 ACDVs retained perforin, granzyme and FasL. Based on their ease of production and lower cost, we chose NK92 ACDVs for in vivo and ex vivo studies. Intravenously injected nimotuzumab‐conjugated NK92 ACDVs decreased the tumour volumes of EGFR‐expressing ovarian cancer xenografts in mice. 14F7hT‐conjugated NK92 ACDVs showed cytotoxic activity against chronic lymphocytic leukaemia biopsies.

This research shows the potential for using antibody‐conjugated, cytotoxic T and NK ACDVs as a feasible and effective approach for tumour‐targeted immunotherapy.

## 1 Introduction

Artificial cell‐derived vesicles (ACDVs) derived from immune cells represent a cutting‐edge approach in cancer therapy that harnesses the body's own defense mechanisms to target and eliminate cancer cells (Seo et al. 2018; Shin et al. 2022; Jong et al. 2017; Wu et al. 2019). CD8+ cytotoxic T cells and natural killer (NK) cells are integral to the immune system's response against cancers, primarily employing two potent mechanisms to eradicate cancer cells: the perforin/granzyme pathway and the Fas ligand (FasL) and TRAIL pathway (Nakajima and Henkart 1994; Lopez et al. 2013; Pardo et al. 2002; Kreuwel et al. 1999; Oshimi et al. 1996; Anel et al. 1995; Mirandola et al. 2004; Takeda et al. 2001). The perforin/granzyme pathway involves granules that contain perforin, a protein that forms pores in the cell membrane, and granzymes, proteases that enter through these pores to initiate apoptosis within cells. The FasL and TRAIL pathway utilizes the binding of FasL and TRAIL ligands on T and NK cells to the Fas and TRAIL receptors on cancer cells, triggering apoptosis through a cascade of intracellular signalling (Kreuwel et al. 1999; Knight et al. 2001; Gagnoux‐Palacios et al. 2018; Takeda et al. 2002; Afshar‐Sterle et al. 2014; Straus et al. 2001; Dorothée et al. 2002). By integrating these natural cytotoxic molecules into the design of ACDVs, we can create biocompatible, targeted therapeutics (Zhu et al. 2018; Hong et al. 2021).

Biologically engineered nanovesicles (NVs), sourced from T and NK cells, encapsulate natural cytotoxic molecules, including perforin, granzymes, and other immune‐modulating factors (Seo et al. 2018; Wu et al. 2019; Zhu et al. 2018; Hong et al. 2021). The unique ability of NVs to mimic their parent immune cell properties allows them to specifically destroy cancer cells (Zhu et al. 2018; Hong et al. 2021).

The application of NVs as cancer therapeutics leverages their inherent biocompatibility, reduced immunogenicity, and the capacity to cross biological barriers like the blood–brain barrier and tumour microenvironment (TME), addressing many challenges in current cancer treatment modalities (Crum et al. 2023; Ou et al. 2022; Yu et al. 2022; Morad et al. 2019). Furthermore, the versatility of NVs can be enhanced through genetic and surface modifications, and loading with chemotherapeutic drugs, allowing for the precise targeting of cancer cells while minimizing off‐target effects and toxicity (Yu et al. 2022; Ruan et al. 2023; Ohno et al. 2013; Fu et al. 2019; Jung et al. 2022; Tao et al. 2023; Smyth et al. 2014).

While the therapeutic benefits of natural immune cell‐derived NVs are significant, producing them in quantities sufficient for clinical needs is challenging due to the complex and costly isolation and purification processes (Jong et al. 2017; Oh et al. 2015). Therefore, ACDVs derived from immune cells, which can be produced in greater yields, represent a promising alternative (Zhu et al. 2018; Hong et al. 2021; Oh et al. 2015). To enhance the cytotoxic capacity of natural cell‐derived NVs, targeted versions are being created from genetically engineered immune cells, expressing chimeric antigen receptors and peptides or through surface modifications using copper‐free click chemistry techniques (Ruan et al. 2023; Ohno et al. 2013; Fu et al. 2019; Jung et al. 2022; Tao et al. 2023; Smyth et al. 2014; He et al. 2022).

Strain‐promoted azide‐alkyne cycloaddition (SPAAC) reaction, a form of copper‐free click chemistry, has gained popularity in cell engineering and bioconjugation due to its specificity, high reaction rate, small molecular size, and biocompatibility (Smyth et al. 2014; Devaraj and Finn 2021; Takayama et al. 2019). The lysine residues on NVs surface proteins can be modified with azide moieties and conjugated to dibenzocyclooctyne (DBCO)‐labelled antibodies through SPAAC, enhancing targeting capabilities (Ruan and Zhao 2023).

Here, we describe the production, characterization, and comparison of ACDVs derived from activated‐T cells and NK92 cells conjugated with tumour‐targeting antibodies, nimotuzumab and 14f7hT. Nimotuzumab, a humanized monoclonal antibody, has been developed for treating various cancers that express epidermal growth factor receptor (EGFR), including glioma, squamous cell carcinoma of the head and neck, colorectal, non‐small cell lung cancer and breast cancer (Ramakrishnan et al. 2009; Mazorra et al. 2018). The humanized monoclonal antibody 14f7hT targets N‐glycolyl neuraminic acid (Neu5Gc) GM3 ganglioside, which is a foreign antigen that is readily taken up by a variety of cancer cells (Fernández‐Marrero et al. 2011; Samraj et al. 2014; Malykh et al. 2001).

With the aim of harnessing the potential of ACDVs derived from activated‐T and NK cells as immunotherapeutics, we report a method for displaying tumour‐targeting antibodies on ACDVs derived from activated‐T cells and NK92 cells. We compared properties of antibody‐conjugated, activated‐T cell ACDVs (T‐ACDVs) and NK92 cell ACDVs (NK92‐ACDVs).

## 2 Materials and Methods

### 2.1 Cell Culturing

Primary human T cells were acquired from healthy donors following a protocol approved by the Institutional Review Board. Human peripheral blood mononuclear cells (PBMCs) were separated from whole blood using Ficoll‐Hypaque (Sigma). CD4^+^/CD8^+^ T cells were isolated using a positive magnetic selection kit (StemCell Technologies), maintaining a 1:1 ratio of CD4^+^/CD8^+^. T cells were cryopreserved with 90% fetal bovine serum (FBS) (Sigma) and 10% dimethyl sulfoxide (DMSO) (Sigma). T cells were cultured in ImmunoCult (StemCell Technologies), supplemented with 100 IU/mL human recombinant IL‐2 (StemCell Technologies) and 100 IU/mL penicillin/streptomycin (Gibco). T cells were expanded by culturing for 6 days with ImmunoCult CD3/CD28 T cell activator reagent (StemCell Technologies) administered on Days 1 and 5.

Cell lines were purchased from the American Type Culture Collection (ATCC). Cells were cultured in media with 100 IU/mL penicillin/streptomycin in a 5% CO_2_ at 37°C. NK‐92 cells were cultured in Alpha Minimum Essential Medium (Gibco) with 12.5% FBS (Gibco), 12.5% horse serum (Gibco), 100 IU/mL IL‐2 (StemCell Technologies), 0.2 mM Myo‐inositol (Sigma), 0.1 mM 2‐mercaptoethanol (Gibco) and 0.02 mM folic acid (Sigma). L1210 cells were cultured in Dulbecco's Modified Eagle Medium (DMEM) with 10% horse serum (Gibco). SK‐OV‐3 cells were cultured in Roswell Park Memorial Institute (RPMI) 1640 Medium with 10% FBS (Gibco).

### 2.2 Animals

NOD scid gamma (NSG) female mice (Jackson lab), aged 4–6 weeks, were housed following the University Animal Care Committee (UACC) guidelines. All experiments and euthanasia were performed in accordance with UACC guidelines.

### 2.3 Labelling of Antibody With DBCO‐PEG_4_‐NHS Ester

Nimotuzumab and 14f7hT antibodies were supplied by the Centre of Molecular Immunology (CIM) (Havana, Cuba). Antibodies were labelled non‐specifically with DBCO‐PEG_4_‐NHS ester (Click Chemistry Tools) using a molar ratio of 1:3 antibody to DBCO labelling reagent. The reaction was incubated at room temperature (RT) with shaking (600 rpm) for 2 h. Unreacted DBCO‐PEG_4_‐NHS ester was removed using a Zeba Spin 40 kDa molecular weight cut‐off desalting column (Thermo Fisher Scientific). A_280_ and A_309_ were measured using a NanoDrop 2000c spectrophotometer (Thermo Fisher Scientific) to determine the protein concentration and degree of labelling.

### 2.4 Production and Characterization of Antibody‐Conjugated T‐ and NK‐92 Artificial Cell Derived Vesicles by Ultrasonication

Antibody‐conjugated T‐ACDVs and NK92‐ACDVs were synthesized in two steps. Initially, a suspension of immune cells (20 million/mL in PBS) was sonicated three times in an ice bath using a Fisher sonicator set to 70% amplitude and 100 W power with a 1 s on/off pulse for 2.5 min, spaced by 5 min intervals. The sonicated cell suspension was centrifuged at 16,000 ×* g* for 20 min at 4°C, and the supernatant containing crude nanovesicles (crude‐ACDVs) was collected. Crude‐ACDVs were treated with 500 µM of 6‐azidohexanoic acid NHS ester (NHS‐Az) for 30 min at RT, followed by purification using an ultracentrifuge (UCF) (rotor type: SW 41Ti, Beckman Coulter) to produce azide‐labelled nanovesicles (ACDV‐Azs). During the ultracentrifugation, a density gradient of 50% and 10% iodixanol was used, and the crude‐ACDV‐Az was layered on top of the iodixanol gradient in a UCF tube. The sample was centrifuged at 100,000 ×* g* for 2 h at 4°C. ACDV‐Azs were obtained from the layer between 10% and 50% iodixanol.

In the second step, ACDV‐Azs were conjugated with DBCO‐nimotuzumab or DBCO‐14f7hT (4 µM) at 37°C for 2 h, followed by 10× dilution with PBS. The antibody‐labelled ACDVs (T‐ACDV‐nimotuzumab, T‐ACDV‐14f7hT, NK92‐ACDV‐nimotuzumab and NK92‐ACDV‐14f7hT) were purified using ultracentrifugation (100,000 ×* g* for 2 h at 4°C) with a density gradient of 50% and 10% iodixanol. The antibody‐labelled ACDVs were obtained from the layer between the 10% and 50% iodixanol. We obtained approximately ∼0.5 mg of T‐ACDVs and ∼1.5 mg of NK92‐ACDVs as measured by bicinchoninic acid assay (BCA) (Thermo Fisher Scientific).

The conjugation of DBCO‐antibody to the surface of ACDV‐Azs was verified by mixing 50 µg of antibody‐labelled ACDVs with 50 µL of 1:200 PBS‐diluted aldehyde/sulfate‐latex beads (diameter = 4 µm; 5.5 × 10^6^ particles/mL; Invitrogen, Carlsbad, CA) and incubating for 15 min at RT. The bead mixture was then diluted to 1 mL with PBS and shaken at 600 rpm for 2 h at RT. The bead‐immobilized ACDVs were centrifuged at 5000 ×* g* for 5 min and blocked with fetal bovine serum (FBS) for 30 min at RT. The FBS‐blocked, bead‐coated ACDVs were centrifuged at 5000 ×* g* for 5 min, stained with PE mouse anti‐human IgG (BD Biosciences), washed with PBS, and subsequently analysed by flow cytometry.

### 2.5 Dynamic Light Scattering (DLS)

ACDVs hydrodynamic size was determined using a Zetasizer Ultra Red (Malvern Panalytical). An aqueous dispersion of ACDVs was prepared at 0.5 mg/mL. Size‐distribution readings were derived from three measurements. Each measurement had a minimum of ten individual runs. The resulting data were reported as size distribution by intensity (percent) and polydispersity index (PI).

### 2.6 Transmission Electron Microscopy (TEM)

For negative staining, 5 µL of ACDVs were placed on a freshly glow discharged 400 mesh Cu grid coated with formvar and carbon. ACDVs adhered to the surface for 2 min. The grid was washed with a drop of distilled water for 20 s and then stained with 2% Uranly Acetate for 1 min. Grids were imaged using a TEM HT7700 (Hitachi High‐Tech).

### 2.7 Perforin and Granzyme B Expression

The presence of perforin and granzyme B in T‐ACDVs and NK92‐ACDVs was verified by mixing 50 µg of ACDVs with 50 µL of 1:200 PBS‐diluted aldehyde/sulfate‐latex beads (diameter = 4 µm; 5.5 × 10^6^ particles/mL; Invitrogen, Carlsbad, CA) and incubating for 15 min at RT. The mixture was then diluted to 1 mL with PBS and shaken at 600 rpm for 2 h at RT. The bead‐immobilized ACDVs were centrifuged at 5000 ×* g* for 5 min and blocked with fetal bovine serum (FBS) for 30 min at RT. The FBS‐blocked, bead‐immobilized ACDVs were centrifuged at 5000 ×* g* for 5 min, resuspended with 100 µL of fixation buffer (eBioscience) and incubated for 30 min at RT. The fixed beads were resuspended with 1 mL of 1× permeabilization buffer (eBioscience), centrifuged at 5000 ×* g* for 5 min, stained with FITC anti‐human perforin antibody (Biolegend) and APC anti‐human/mouse granzyme B antibody (Biolegend) in 1× permeabilization buffer for 1 h. The beads were washed with PBS, resuspended in PBS and analysed by flow cytometry.

### 2.8 FasL and TRAIL Expression

The presence of Fas and TRAIL ligands on the surface of T‐ACDVs and NK92‐ACDVs was verified by mixing 5 µg of ACDVs with 50 µL of 1:200 PBS‐diluted aldehyde/sulfate‐latex beads (diameter = 4 µm; 5.5 × 10^6^ particles/mL; Invitrogen, Carlsbad, CA) and incubating for 15 min at RT. The mixture was then diluted to 1 mL with PBS and shaken at 600 rpm for 2 h at RT. Bead‐immobilized ACDVs were centrifuged at 5000 ×* g* for 5 min and blocked with fetal bovine serum (FBS) for 30 min at RT. FBS‐blocked, bead‐immobilized ACDVs were centrifuged at 5000 ×* g* for 5 min, stained with APC anti‐human CD178 (Fas‐L) antibody (Biolegend) and PE anti‐human CD253 (TRAIL) antibody (Biolegend), washed with PBS, and analysed by flow cytometry.

### 2.9 Expression of GM3 (Neu5Gc) in L1210 and EFGR in SK‐OV‐3 Cells

The expression of GM3 (Neu5Gc) and EGFR on the surface of L1210 and SK‐OV‐3 cells, respectively, was verified by mixing 14f7hT and nimotuzumab with cancer cell lines (5 × 10^4^ cells) at 100 nM in a 96‐well flat‐bottom plate in a total volume of 100 µL RPMI with 10% FBS. Cells were incubated for 2 h at 37°C, washed with PBS, stained with FITC Goat anti‐(H+L chain) antibody (Novus Biologicals), washed with PBS, and analysed by flow cytometry.

### 2.10 Cell Interaction Assay

Azide‐modified T‐ACDVs or NK92‐ACDVs were labelled with 5 µL Vybrant DiD Cell‐Labelling Solution (DiDR, Thermo Fisher Scientific), according to the manufacturer's instructions. Unincorporated DiDR was removed using the UCF purification procedure described above. DBCO‐labelled antibody was conjugated to the DiDR‐stained ACDV‐Azs and purified using the UCF purification procedure. DiDR‐stained T‐ACDVs or NK92‐ACDVs or antibody‐conjugated T‐ACDVs or NK92‐ACDVs were added to cancer cell lines (5 × 10^4^ cells) at 50 µg/mL in a 96‐well flat‐bottom plate in a total volume of 100 µL PBS. Cells were incubated for 2 h at 37°C and analysed by flow cytometry.

### 2.11 Cytotoxicity Assay

For L1210 cells, 50 µg/mL of 14f7hT conjugated and unconjugated T‐ACDVs or NK92‐ACDVs were mixed with 5 × 10^4^ cancer cells for 16 h at 37°C, stained with 7‐AAD cell viability dye (Thermo Fisher Scientific), and analysed by flow cytometry. The percent cell death was determined by analysing 7‐AAD positive L1210 cells.

SK‐OV‐3 cells expressing green fluorescent protein (GFP) were cultured in RPMI‐1640 medium supplemented with 10% FBS and 1 mg/mL zeocin (Invitrogen) under standard conditions (37°C, 5% CO_2_). Live‐cell imaging was performed using an IncuCyte S3 Live‐Cell Analysis System (Sartorius). SK‐OV‐3 GFP cells were seeded in 96‐well plates at 2500 cells per well and allowed to adhere overnight. Cells were treated with 50 µg/mL of nimotuzumab conjugated or unconjugated T‐ACDVs or NK92‐ACDVs. Cells were imaged every 4 h using a 10× objective for 48 h. GFP fluorescence intensity was measured to assess cell viability. Data were processed using IncuCyte software to quantify GFP expression. The measured fluorescence was normalized, and the area under the curve (AUC) was calculated.

### 2.12 Internalization Assay

T‐ACDVs and NK92‐ACDVs were treated with pHAb amine‐reactive dye (Promega) at 10 µM for 30 min at RT, and then with 500 µM NHS‐Az for 30 min at RT. Unbound dye and NHS‐Az were removed by the UCF purification procedure described above. The pHAb‐labelled ACDV‐Azs were conjugated to nimotuzumab‐DBCO and purified using the UCF purification as described previously. Nimotuzumab conjugated or unconjugated pHAb‐labelled T‐ACDVs or NK92‐ACDVs were added to the SK‐OV‐3 cells (5 × 10^4^ cells) at 50 µg/mL in a 96‐well flat‐bottom plate in a total volume of 100 µL RPMI‐1640 and 10% FBS and incubated under 5% CO_2_ at 37°C. The internalization of ACDVs was measured as a change in MFI by flow cytometry at 0, 0.5, 2.5 and 4.5 h.

### 2.13 In Vivo Cytotoxicity of NK92‐NV‐Nimotuzumab in SK‐OV‐3 Xenograft NOD Scid Gamma (NSG) Mouse Model

For xenograft preparation, 5 × 10^6^ SK‐OV‐3 cells were collected and washed with RPMI medium without FBS. Cells were suspended in 50 µL of RPMI medium and 50 µL of Matrigel membrane matrix (Corning, Corning, NY) and injected subcutaneously into the right hind flank of the 4–6 week‐old NSG mice. Tumour sizes were monitored every 2 days until they reached 100.2 ± 33.63 mm^3^. Mice bearing SK‐OV‐3 xenografts were divided into three groups (n ≥ 5 mice/group): (i) NK92‐ACDV‐Nimotuzumab (six doses of 100 µg/dose), (ii) NK92‐ACDV (six doses of 100 µg/dose) and (iii) PBS control. All treated mice received six doses intravenously via the tail vein on Days 0, 2, 4, 6, 8 and 10. Tumour growth was monitored by measuring the greatest length and width using a digital calliper (tumour volume = (length × width^2^)/2). The study was terminated when tumours reached a volume ≥ 1000 mm^3^ or on Day 22. These volumes were used to determine survival in the different groups using Kaplan–Meier curves. The body weight of each mouse was recorded every other day during the experimental period.

### 2.14 Chronic Lymphocytic Leukaemia Patients Cell Cytotoxicity

PBMCs were obtained from patients diagnosed with chronic lymphocytic leukaemia (CLL) based on the World Health Organization criteria (Weltgesundheitsorganisation 2017). PBMCs from CLL patients were collected under an Institutional Review Board‐approved protocol. The cryopreserved CLL cells vial was thawed, and CLL cell viability was assessed by trypan blue exclusion. The viability of CLL cells (∼90%) was used in the cytotoxicity assay. CLL cells were cultured in RPMI medium supplemented with 10% FBS and 100 IU/mL IL‐2 for 1 h. After 1 h, NK92‐ACDVs and NK92‐ACDV‐14f7hT were mixed with 1 × 10^5^ cells at a concentration of 50 µg/mL and incubated for 16 h. To identify the 14f7hT positive population among CLL cells, cells were stained with biotinylated 14f7hT for 30 min at RT, followed by staining with Streptavidin FITC conjugate (Thermo Fisher Scientific) for 30 min on ice. Cells were stained with 7‐AAD and CLL cell death was assessed by analysing 7‐AAD staining on 14f7hT positive cancer cells.

### 2.15 Statistical Analysis

Data are displayed as the mean ± standard deviation with a sample size (n) for statistical analysis of at least three. Statistical analyses were performed using Student's *t*‐test, ordinary one‐way ANOVA (Tukey's multiple comparisons test), 2‐way ANOVA (Šídák's multiple comparisons test), and Log‐rank (Mantel‐Cox) test. *p* values of *<0.05, **<0.01, ***<0.001 and ****<0.0001 were considered statistically significant, ns, no statistically significant difference.

## 3 Results

### 3.1 Production and Characterization of Antibody‐Conjugated Artificial Cell‐Derived Vesicles From Activated‐T Cells and NK92 Cells

**FIGURE 1:**
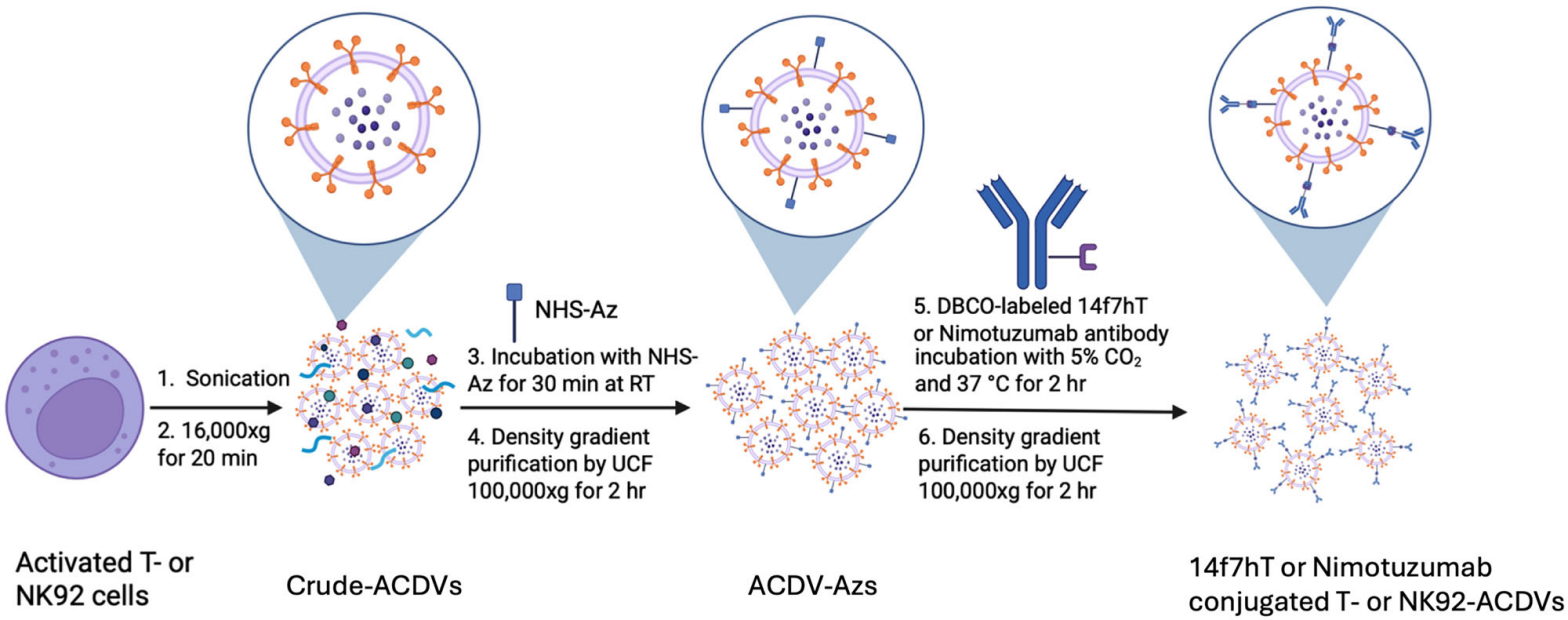
Production of antibody‐conjugated artificial cell‐derived vesicles from immune cells (Seo et al. 2018). Activated‐T cells and NK92 cells are sonicated to produce crude nanovesicles (crude‐ACDVs) (Shin et al. 2022). Large contaminants are removed by centrifugation (Jong et al. 2017). Crude‐ACDVs are labelled with azides by reacting with 6‐azidohexanoic acid NHS ester (NHS‐Az) (Wu et al. 2019). Unreacted NHS‐Az and small contaminants are removed by density gradient ultracentrifugation (UCF) (Nakajima and Henkart 1994). Azide‐modified ACDVs (ACDV‐Azs) are reacted with a dibenzocyclooctyne (DBCO)‐containing antibody via the SPAAC reaction to produce antibody‐conjugated ACDVs (Lopez et al. 2013). Antibody‐conjugated T‐ or NK92‐ACDVs are purified using density gradient UCF. Created in BioRender. Parlekar, B. (2025) https://BioRender.com/u28m085.

We generated ACDVs using activated‐T cells and NK92 cells. Activated‐T cells were produced by culturing T cells with anti‐CD3/CD28 antibody activation beads and expanding them for 6 days in ImmunoCult medium supplemented with recombinant IL‐2. NK92 cells are a clinically relevant NK cell line, which shows characteristics of highly active natural killer cells and can be engineered and given to patients with cancer (Wang et al. 2017; Liu et al. 2020; Tang et al. 2018). We modified nimotuzumab or 14f7hT antibodies with DBCO by conjugating them with DBCO‐PEG_4_‐N‐hydroxysuccinimidyl ester (DBCO‐PEG_4_‐NHS ester), resulting in 1–2 DBCO molecules per antibody. We generated antibody‐modified, activated‐T cell ACDVs (T‐ACDVs) and NK92 cell ACDVs (NK92‐ACDVs) using a two‐step process (Figure 1). First, we sonicated activated‐T cells or NK‐92 cells (20 million cells/mL in PBS) three times to generate crude ACDVs. Large contaminants were removed by centrifugation at 16,000 ×* g*. We modified the crude mixture of ACDVs with azide moieties by reacting them with 6‐Azidohexanoic acid N‐hydroxysuccinimide ester (NHS‐Az). We purified the azide‐modified ACDVs (ACDV‐Az) using ultracentrifugation (UCF) with an iodixanol gradient to remove unreacted NHS‐Az and small contaminants. Second, we conjugated DBCO‐modified antibodies (nimotuzumab or 14f7hT) to the purified ACDV‐Azs using the SPAAC reaction. We further purified the antibody‐conjugated ACDVs using UCF with an iodixanol gradient.

**FIGURE 2:**
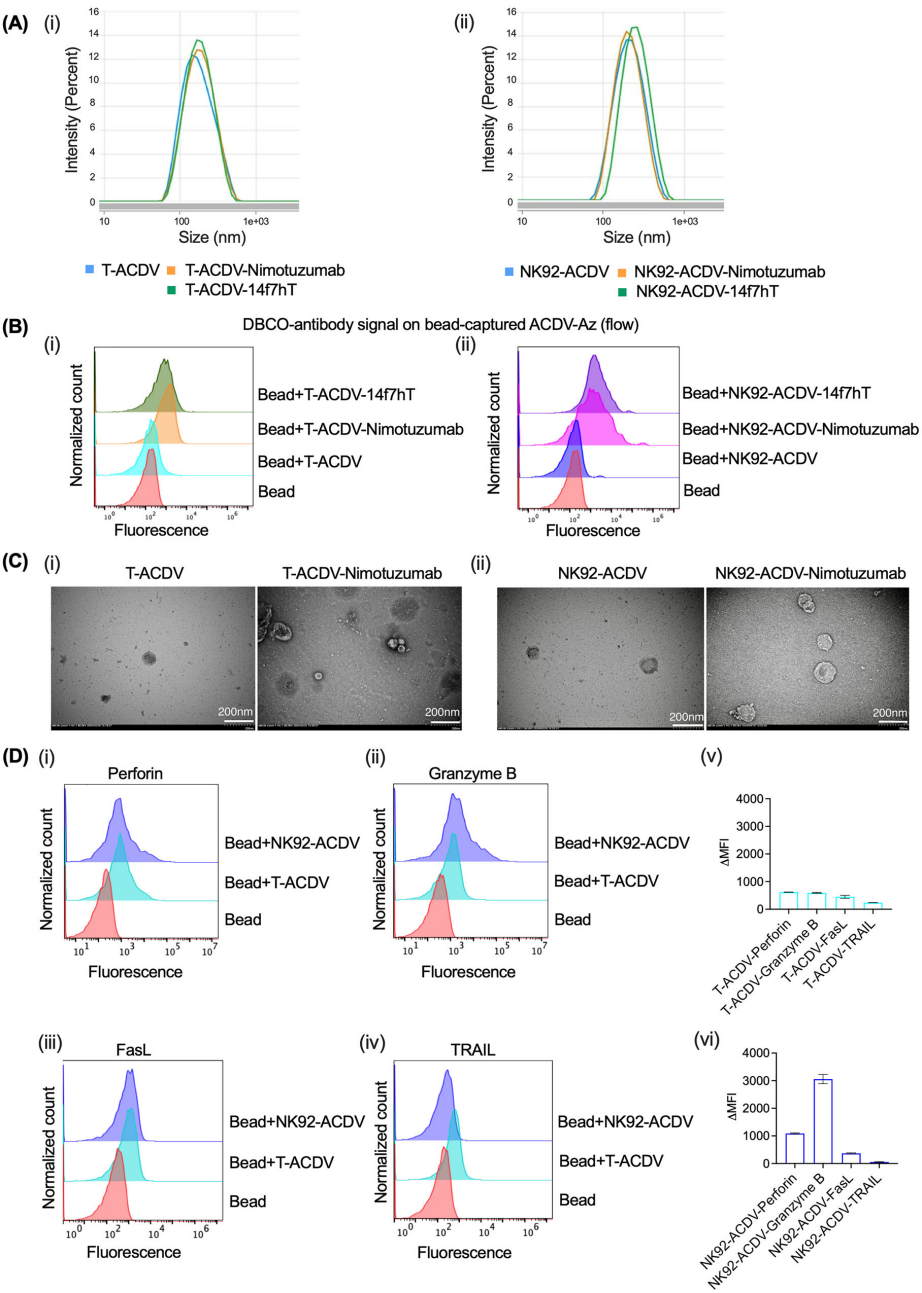
Characterization of Nimotuzumab‐ and 14f7hT‐conjugated T‐ACDVs and NK92‐ACDVs. (A) Size distribution of antibody conjugated and unconjugated T‐ACDVs (i) and NK92‐ACDVs (ii) calculated by dynamic light scattering. (B) Analysis of DBCO‐antibody conjugation to azide‐modified T‐ACDV (T‐ACDV‐Az) (i) and azide‐modified NK92‐ACDV (NK92‐ACDV‐Az) (ii) using flow cytometry with bead immobilized ACDVs. (C) Representative transmission electron microscopy image of nimotuzumab conjugated and unconjugated T‐ACDVs (i) and NK92‐ACDVs (ii) (scale bars denote 200 nm). (D) Measurement of perforin (i), granzyme B (ii) FasL (iii) and TRAIL (iv) in T‐ACDVs and NK92‐ACDVs using flow cytometry with bead immobilized ACDVs. (v–vi) T‐ACDVs and NK92‐ACDVs were gated for fluorescence positivity, and the change in mean fluorescence intensity (ΔMFI) relative to beads was reported.

We characterized antibody‐conjugated and unconjugated T‐ACDVs or NK92‐ACDVs using dynamic light scattering (DLS) and transmission electron microscopy (TEM). Antibody conjugated and unconjugated ACDVs were dispersed at 0.5 mg/mL in PBS. The hydrodynamic diameter of unconjugated T‐ACDVs and NK92‐ACDVs was 188.3 and 171.1 nm, respectively. The hydrodynamic diameter of antibody‐conjugated ACDVs ranged from 198.1 to 258.8 nm. T‐ACDV‐Nimotuzumab (201.1 nm), T‐ACDV‐14f7hT (213.1 nm), NK92‐ACDV‐Nimotuzumab (258.8 nm) and NK92‐ACDV‐14f7hT (198.1 nm) (Figure 2A). The polydispersity index (PI) for antibody conjugated and unconjugated T‐ACDVs and NK92‐ACDVs was less than 0.5 nm.

Antibody conjugation to the T‐ACDV and NK92‐ACDV was confirmed using flow cytometry. Due to their small size, ACDVs need to be immobilized on larger 4 µm aldehyde/sulfate‐latex beads to be detected by flow cytometry. Antibodies conjugated on the bead‐bound ACDVs were detected using a fluorescent secondary antibody (Figure 2B).

The TEM image of antibody‐conjugated and unconjugated T‐ and NK92‐ACDVs revealed A similar unilamellar structure with a lipid bilayer, indicating that the conjugation did not affect the ACDVs’ structure or integrity (Figure 2C).

Perforin and granzyme are highly expressed in activated‐T cells and NK92 cells (Lieberman 2003). To determine whether these molecules were retained in T‐NVs and NK92‐NVs, we detected their presence in ACDVs using flow cytometry (Figure 2D). T‐ACDVs and NK92‐ACDVs were first bound to latex beads, and then intracellular fixation and permeabilization buffer were used to detect perforin and granzyme B, which were stained with anti‐human perforin and anti‐human/mouse granzyme B antibodies. Both T‐ACDVs and NK92‐ACDVs retained granzyme B and perforin.

Apoptosis inducing transmembrane proteins such as FasL and TRAIL ligands are highly expressed in activated‐T cells and NK92 cells (Mirandola et al. 2004). We detected FasL and TRAIL on T‐ACDVs and NK92‐ACDVs using flow cytometry. T‐ACDVs and NK92‐ACDVs were bound to latex beads, and FasL and TRAIL ligands were detected using anti‐FasL and anti‐TRAIL antibodies. T‐ACDVs retained Fas and TRAIL ligands, whereas NK92‐ACDVs retained FasL only (Figure 2D).

### 3.2 Specific Binding of 14f7hT and Nimotuzumab Conjugated T‐ACDVs or NK92‐ACDVs to Antigen‐Positive Cancer Cells

**FIGURE 3:**
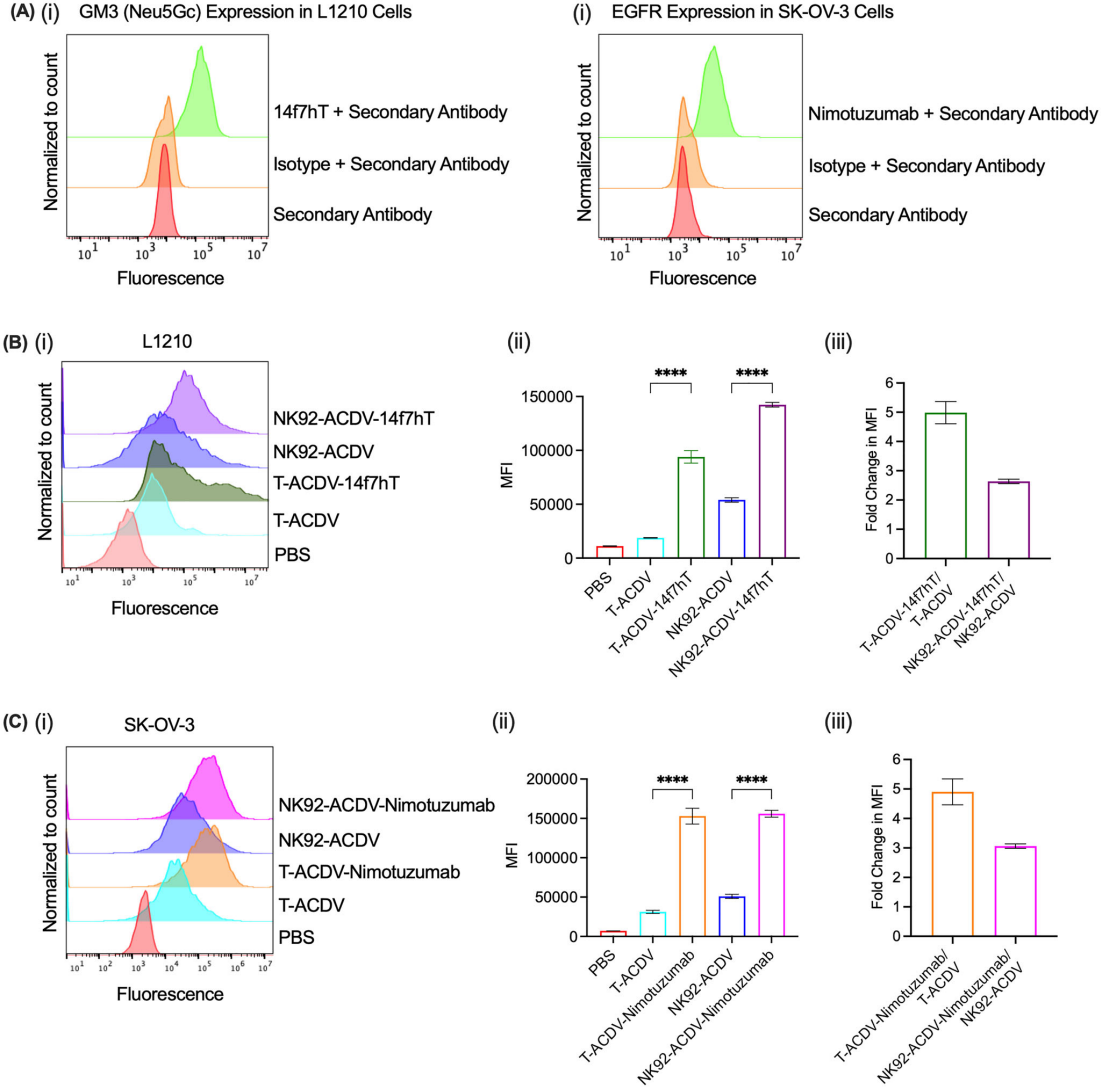
Interaction of antibody conjugated T‐ACDVs and NK92‐ACDVs with antigen positive cells. (A) 14f7hT and nimotuzumab positivity on L1210 (i) and SK‐OV‐3 (ii) cancer cells, respectively. DiDR labelled 14f7hT‐conjugated T‐ACDVs and NK92‐ACDVs (B) and nimotuzumab‐conjugated T‐ACDVs and NK92‐ACDVs (C) were cultured with Neu5Gc GM3 positive (L1210) and EGFR positive (SK‐OV‐3) cells, respectively, and analysed using flow cytometry (i). The mean fluorescence intensity (MFI) is shown for each treatment (ii). The fold change in MFI for T‐ACDV‐antibody relative to T‐ACDV, and NK92‐ACDV‐antibody relative to NK92‐ACDV is shown (iii). Data represent the mean ± SD (*n* = 3). One‐way ANOVA, ****p < 0.0001.

L1210 and SK‐OV‐3 cells were assessed for 14f7hT and nimotuzumab positivity using flow cytometry, respectively (Figure 3Ai,ii). We determined whether the conjugation of antibodies to T‐ACDVs and NK92‐ACDVs enhanced their interaction with antigen‐positive cancer cells. 14f7hT or nimotuzumab conjugated and unconjugated ACDVs were stained with Vybrant DiD Cell‐Labelling dye (DiDR) and incubated with Neu5Gc‐GM3 positive L1210 murine lymphocytic leukaemia cells or EGFR‐positive human SK‐OV‐3 ovarian cancer cells. DiDR‐positive cells were detected by flow cytometry. T‐ACDV‐14f7hT and NK92‐ACDV‐14f7hT displayed 4.99 and 2.64‐fold higher interaction with L1210 cancer cells relative to T‐ACDVs and NK92‐ACDVs, respectively (Figure 3Biii). 14f7hT conjugated T‐ACDVs or NK92‐ACDVs displayed significantly higher binding to L1210 cancer cells compared to T‐ACDVs or NK92‐ACDVs (Figure 3Bi,ii).

T‐ACDV‐Nimotuzumab and NK92‐ACDV‐Nimotuzumab displayed 4.90 and 3.06‐fold higher interaction with SK‐OV‐3 cancer cells relative to unconjugated T‐ACDVs or NK92‐ACDVs, respectively (Figure 3Ciii). Nimotuzumab conjugated T‐ or NK92‐ACDVs displayed significantly higher binding to SK‐OV‐3 cancer cells compared to T‐ and NK92‐ACDVs, respectively (Figure 3Ci,ii).

### 3.3 In Vitro Cytotoxicity of Antibody‐Conjugated T‐ACDVs and NK92‐ACDVs

**FIGURE 4:**
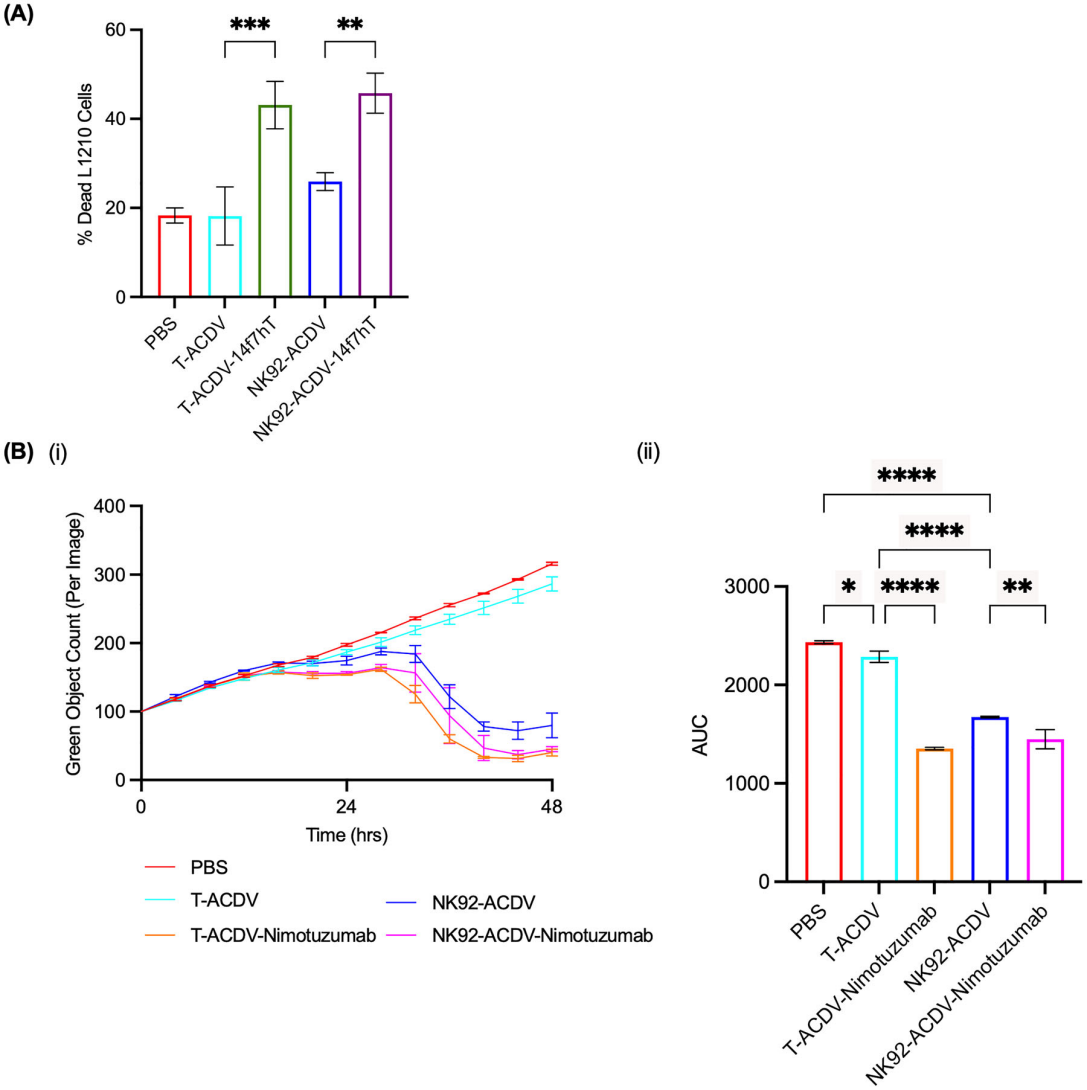
In vitro cytotoxicity of antibody‐conjugated T‐ACDVs or NK92‐ACDVs. (A) 14f7hT‐conjugated or unconjugated T‐ACDVs and NK92‐ACDVs were cultured with L1210. L1210 cells were stained with 7‐AAD and analysed by flow cytometry to quantify the percentage of dead cells. (B) Nimotuzumab‐conjugated and unconjugated T‐ACDVs and NK92‐ACDVs were cultured with SK‐OV‐3 expressing GFP (i). GFP fluorescence intensity was measured to assess cell viability. Data were processed using IncuCyte software to quantify GFP expression. The measured fluorescence was normalized, and the area under the curve (AUC) calculated (ii). Data represent the mean ± SD (*n* = 3). One‐way ANOVA, *p < 0.05, **p < 0.01, ***p < 0.001, ****p < 0.0001.

We examined the in vitro cytotoxicity of T‐ACDV‐14f7hT or NK92‐ACDV‐14f7hT against Neu5Gc‐GM3 positive murine lymphocytic leukaemia cell line, L1210, relative to untargeted T‐ACDVs or NK92‐ACDVs. Antibody‐conjugated or unconjugated T‐ACDVs or NK92‐ACDVs were co‐cultured with L1210 cells, and the percentage of dead L1210 cells was evaluated after 16 h of incubation by staining cancer cells with 7‐AAD dye and analysing by flow cytometry. T‐ACDV‐14f7hT and NK92‐ACDV‐14f7hT showed similar cytotoxicity against L1210 cancer cells, killing 43.1% and 45.8% of L1210 cells, respectively. Unconjugated T‐ACDVs and NK92‐ACDVs displayed lower levels of killing of L1210 cells, 18.2% and 25.9%, respectively, which is similar to PBS treated L1210 cells (18.3%) (Figure 4A).

We evaluated the cytotoxicity of nimotuzumab conjugated T‐ACDVs or NK92‐ACDVs and non‐targeted T‐ACDVs or NK92‐ACDVs against the ovarian cancer cell line, SK‐OV‐3 expressing green fluorescence protein (GFP) using the IncuCyte live cell imager (Figure 4Bi,ii). We observed a significant decrease in SK‐OV‐3 viability when treated with nimotuzumab‐conjugated T‐ACDVs and NK92‐ACDVs compared to unconjugated T‐ or NK92‐ACDVs, respectively, demonstrating target specific killing of cancer cells. The unconjugated NK92‐ACDVs showed only slightly less cytotoxicity than the NK92‐ACDV‐Nimotuzumab, whereas there was a larger difference in cytotoxicity between the T‐ACDVs and T‐ACDV‐Nimotuzumab. Both unconjugated T‐ or NK92‐ACDVs showed significant killing of SK‐OV‐3 cells relative to the untreated control PBS, however the cytotoxicity of NK92‐ACDVs was significantly higher. There was no difference in killing between targeted T‐ACDVs and targeted NK92‐ACDVs.

### 3.4 Internalization of Nimotuzumab Conjugated and Unconjugated T‐ACDVs and NK92‐ACDVs

**FIGURE 5:**
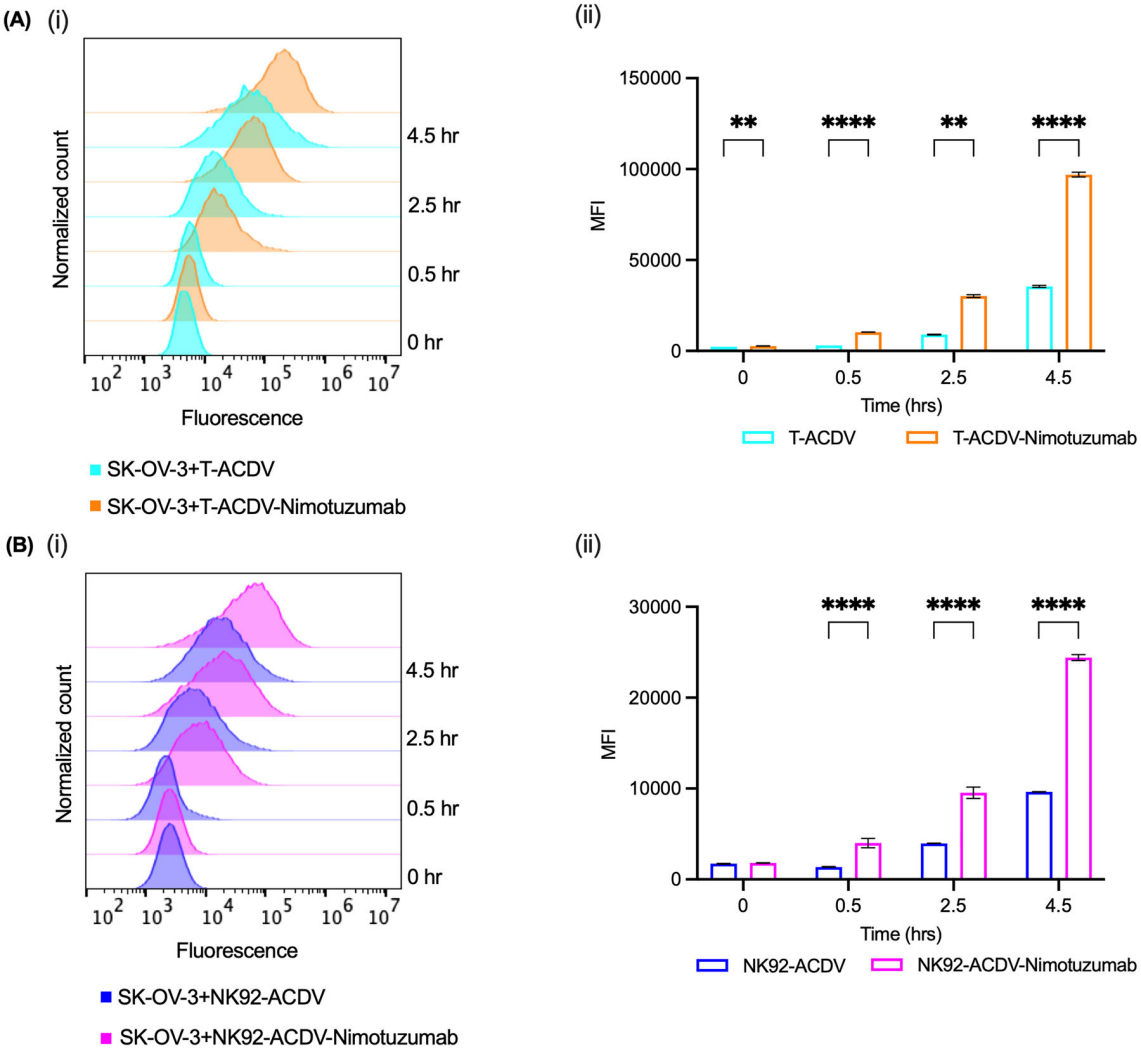
Internalization of nimotuzumab conjugated and unconjugated T‐ and NK92‐ACDVs in SK‐OV‐3. Nimotuzumab conjugated and unconjugated T‐ACDVs (A) and NK92‐ACDVs (B) labelled with pHAb were cultured with SK‐OV‐3 for indicated times. (ii) The mean fluorescence intensity (MFI) is shown for each treatment. Data represent the mean ± SD (*n* = 3). 2‐way ANOVA, **p < 0.01, ****p < 0.0001.

We examined the internalization of nimotuzumab conjugated and unconjugated T‐ACDVs or NK92‐ACDVs in the EGFR‐expressing cancer cell line SK‐OV‐3. To detect ACDV cell internalization, we conjugated the pHAb dye to the surface of the ACDVs. Internalization of antibody‐pHAb Dye conjugates results in trafficking to the endosomal and lysosomal vesicles, which are acidic and cause the pHAb Dye to fluoresce. The internalization of pHAb dye‐labelled ACDVs was measured by flow cytometry (Figure 5A,Bi). For both T‐ACDVs and NK92‐ACDVs, the conjugation of nimotuzumab significantly increased the rate of internalization compared to unconjugated T‐ACDVs and NK92‐ACDVs, respectively (Figure 5A,Bii).

### 3.5 In Vivo Efficacy of NK92‐ACDV‐Nimotuzumab in the SK‐OV‐3 Xenograft NSG Mouse Model

**FIGURE 6:**
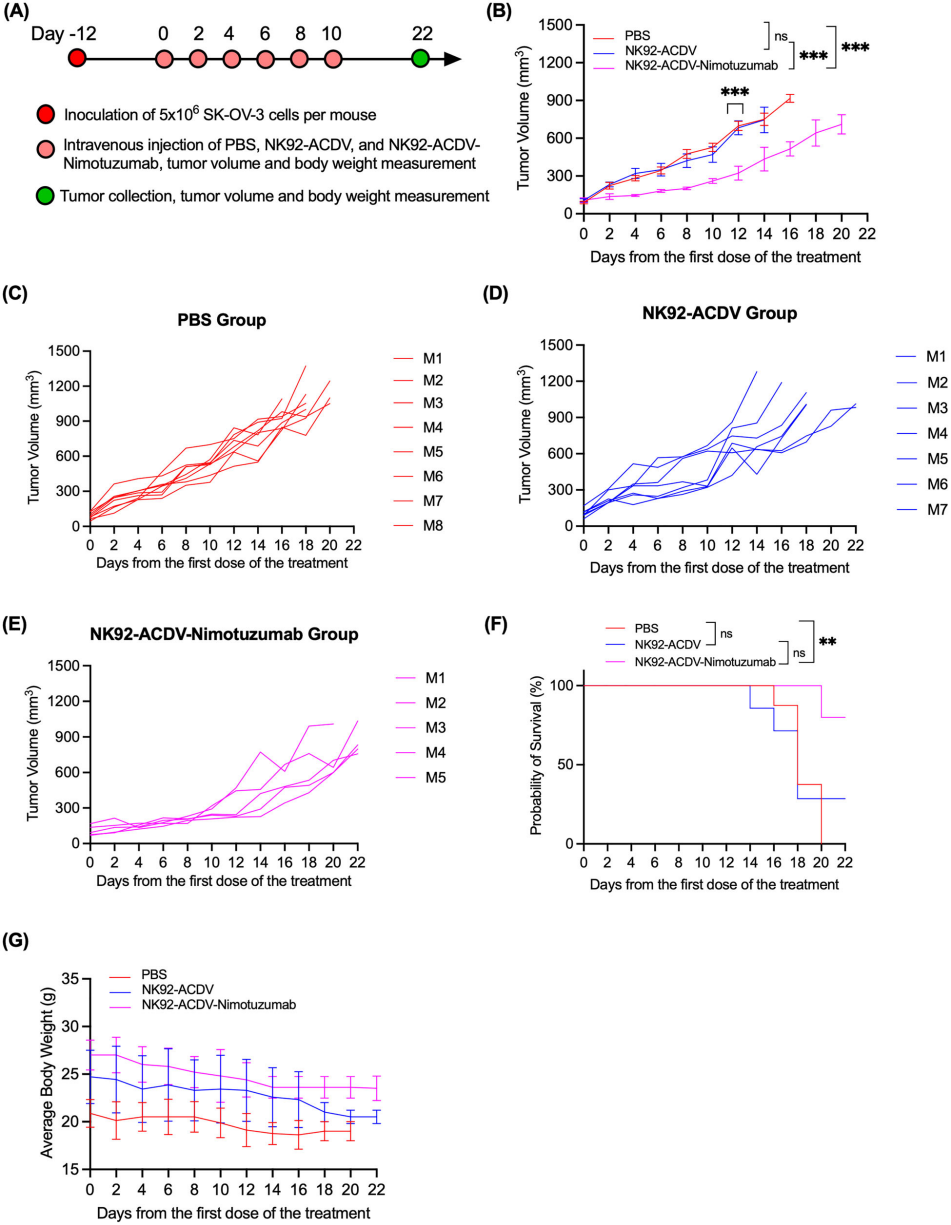
In vivo efficacy of NK92‐ACDV‐Nimotuzumab and NK92‐ACDVs in SK‐OV‐3 xenografts NSG mouse model. (A) Protocol for the in vivo treatment. Mice were treated NK92‐ACDVs, NK92‐ACDV‐Nimotuzumab, and PBS every 2 days for six doses after tumours reached 100.2 ± 33.63 mm^3^ size. The study endpoint was considered as tumour volume ≥ 1000 mm^3^ or survival for 22 days. (B) Average tumour volumes of SK‐OV‐3 xenografts versus time after initial treatment (*n* = 5–8 mice/group). One‐way ANOVA on Day 12, ***p < 0.01, ns, not significant. Individual mouse tumour growth curves for the PBS group (C), NK92‐ACDV group (D), and NK92‐ACDV‐Nimotuzumab group (E). (F) Kaplan–Meier survival curve of mice bearing SK‐OV‐3 xenograft. Log‐rank (Mantel‐Cox) test, **p < 0.01, ns, not significant. (G) Average body weight per group during the treatment period. Data represent the mean ± SD (*n* = 5–8 mice as indicated per group).

We studied the efficacy of NK92‐ACDV‐Nimotuzumab and NK92‐ACDV in a SK‐OV‐3 mouse NSG xenograft model. NSG mice were engrafted with SK‐OV‐3 cells, and after tumours reached the 100.2 ± 33.63 mm^3^ size, mice were treated with 100 µg of ACDVs every 2 days for six doses (Figure 6A). Animals treated with NK92‐ACDV‐Nimotuzumab decreased tumour growth relative to PBS and NK92‐ACDV controls (Figure 6B–E). All the mice in PBS group reached the study endpoint of ≥ 1000 mm^3^ by Day 20. Six of 7 mice (85.7%) treated with non‐targeted NK92‐ACDV reached the study endpoint by Day 22. The Kaplan–Meier survival curve showed that three of 5 mice (60%) treated with NK92‐ACDV‐Nimotuzumab survived until the study endpoint of 22 days (Figure 6F). There was no significant change in the body weight of each mouse throughout the study duration (Figure 6G).

### 3.6 Ex Vivo Efficacy of NK92‐ACDV‐14f7hT in Human Chronic Lymphocytic Leukaemia Biopsies

**FIGURE 7:**
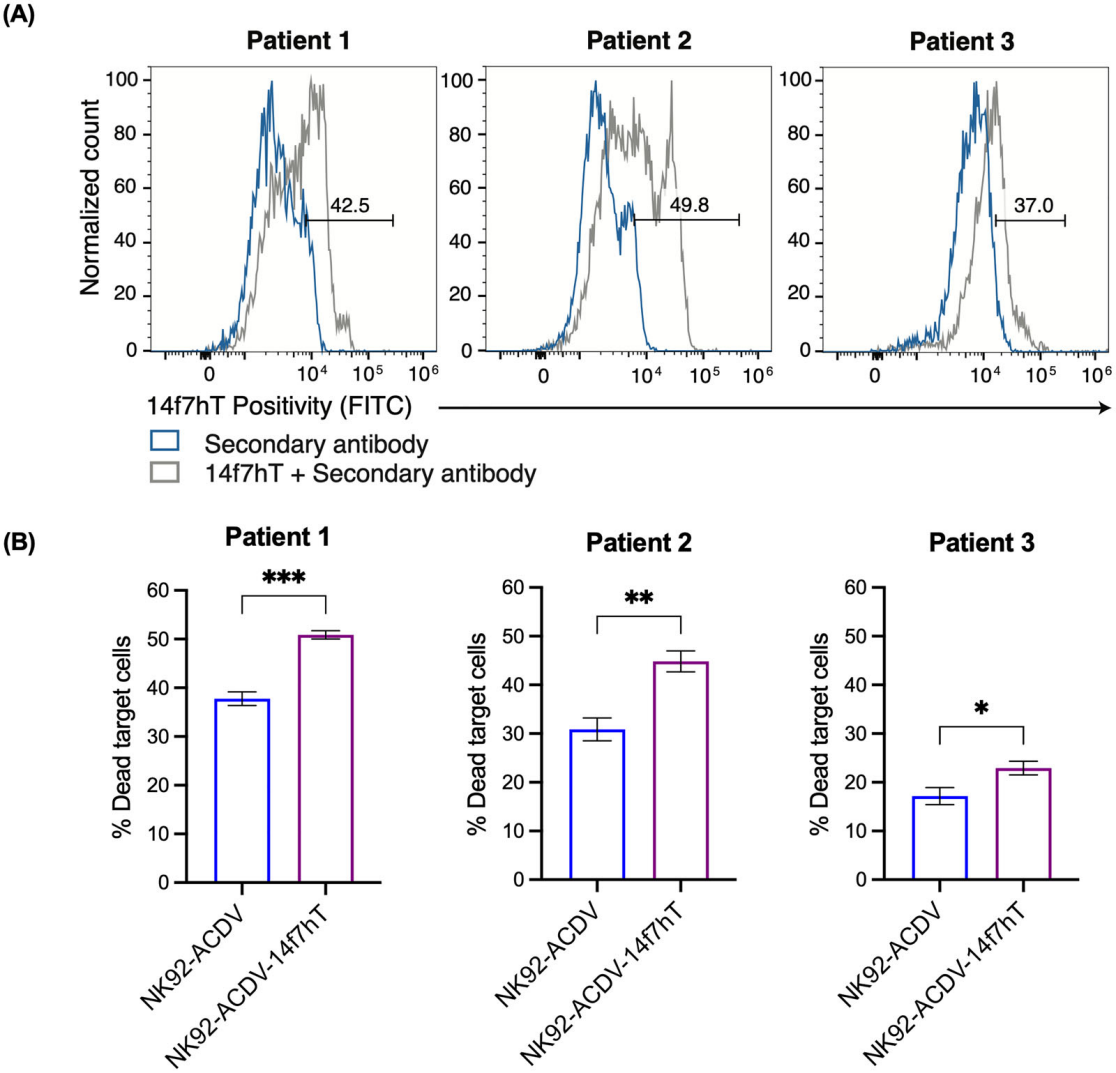
Ex vivo cytotoxicity of 14f7hT‐conjugated and unconjugated NK92‐ACDVs against primary chronic lymphocytic leukaemia cells. (A) 14f7hT positivity in three primary chronic lymphocytic leukaemia (CLL) patient biopsies. (B) 14f7hT‐conjugated and unconjugated NK92‐ACDVs were cultured with primary CLL cells. Cells were stained with 7‐AAD and 14f7hT positive cells were quantified using flow cytometry. Data represent the mean ± SD (*n* = 3). Student's *t*‐test was used for statistical analysis between groups. *p* values of *p < 0.05, **p < 0.01 and ***p < 0.001.

We examined the cytotoxic activity of 14f7hT conjugated NK92‐ACDVs in human CLL patient biopsies. Patient biopsies were assessed for 14f7hT positivity using flow cytometry (Figure 7A). 14f7hT conjugated and unconjugated NK92‐ACDVs were cultured with CLL cells, and cytotoxicity against 14f7hT positive cells was quantified as previously described for L1210 cytotoxicity assay. NK92‐ACDV‐14f7hT had higher levels of killing against 14f7hT‐positive CLL cells relative to unconjugated NK92‐ACDV (Figure 7B).

## 4 Discussion

Immune cell‐derived T‐exosomes or NK‐exosomes can regulate cancer progression and metastasis (Castillo‐Peña and Molina‐Pinelo 2023; Kalluri 2016; Federici et al. 2020). Previous studies reported that exosomes secreted by T and NK cells have an anti‐tumour function *via* inherited cell surface proteins like FasL and TRAIL and encapsulated cytolytic proteins like perforin and granzyme B (Zhu et al. 2018; Hong et al. 2021; Fu et al. 2019; Lugini et al. 2012; Kim et al. 2022). To leverage the cytotoxic abilities of immune cells, we produced ACDVs from activated‐T cells and NK92 cells by ultrasonication. Compared with serial extrusion, ultrasonication offers a filter‐free route to fabricate ACDVs from immune cells, thereby sidestepping practical failures such as polycarbonate‐membrane pore clogging and membrane breakage that can afflict filter‐based workflows and reduce process efficiency (Gao et al. 2023; Van Deun et al. 2020). Sonication‐based protocols have produced ACDVs with higher yields than natural NV secretion (Ng et al. 2022). The performance of ACDVs has also been shown to be of the same order of magnitude as that of extrusion‐derived ACDVs (Ng et al. 2022; Rao et al. 2020). Importantly for immune‐cell applications, sonication produced NVs that preserve source‐cell lipids and membrane proteins—as shown for macrophage‐derived ACDVs—supporting retention of immuno‐functional ligands while avoiding the repeated high‐shear filter passages required by extrusion (Rao et al. 2020; Jang et al. 2023).

ACDVs produced from activated‐T cells and NK cells, which offer a higher yield than natural exosomes (Zhu et al. 2018; Hong et al. 2021), possess tumour‐targeting and killing capabilities. Their small size allows for easy penetration into the TME, and they retain key cell surface proteins, such as Fas and TRAIL, along with cytotoxic proteins like perforin and granzyme B, which enhance their efficacy (Zhu et al. 2018; Hong et al. 2021).

In this study, we describe the conjugation of tumour‐targeting antibodies with T‐ACDVs and NK92‐ACDVs using SPAAC chemistry. Previous studies reported two ways to modify the surface of extracellular vesicles (EVs), which include genetic engineering and chemical modification. Genetic engineering involves combining the gene sequence of a targeting peptide or protein with a specific exosomal membrane protein (He et al. 2022; Bellavia et al. 2017). This method is effective for displaying peptides and proteins on the surface of exosomes and NVs, but it is restricted to targeting motifs that can be encoded genetically and transfected into cells (Liang et al. 2021). Chemical modification enables the display of a broad spectrum of ligands, both natural and synthetic. Chemical conjugation can covalently and stably alter EVs surface proteins without change in size, morphology and function (Smyth et al. 2014; Tian et al. 2018).

We confirmed the size and morphology of antibody‐conjugated ACDVs using DLS and TEM. T‐ACDVs and NK92‐ACDVs were comprised of a lipid bilayer membrane with a diameter of approximately 200 nm, characteristic of immune cell‐derived NVs (Zhu et al. 2018; Hong et al. 2021; Choo et al. 2018). We demonstrated the retention of perforin, granzyme, FasL, and TRAIL in T‐ACDVs, whereas NK92‐ACDVs retained perforin, granzyme, and FasL. Conjugation of antibodies to the surface of T‐ACDVs and NK92‐ACDVs increased their interaction, internalization, and cytotoxicity against cancer cell lines expressing the antibody antigen.

A comparison of untargeted and targeted T‐ACDVs and NK92‐ACDVs showed the following similarities and differences: (i) Both nimotuzumab and 14f7hT‐conjugated NK92‐ACDV and T‐ACDV interacted better with target cells compared to untargeted ACDVs. (ii) Targeted T‐ACDVs and NK92‐ACDVs are internalized better than the corresponding untargeted T‐ACDVs and NK92‐ACDVs in SK‐OV‐3 cells, respectively; in both cases, antibody targeting increases the rate of internalization. (iii) Targeted T‐ACDVs and NK92‐ACDVs have similar cytotoxicity in L1210 and SK‐OV‐3 cells, whereas untargeted NK92‐ACDVs have higher cytotoxicity than untargeted T‐ACDVs, with untargeted NK92‐ACDVs having similar cytotoxicity to targeted NK92‐ACDV and T‐ACDVs in SK‐OV‐3 cells. This indicates that, under our experimental conditions, the presence of an additional death ligand (TRAIL) in T‐ACDVs is not sufficient to enhance cytotoxicity and suggests that other factors—such as differences in vesicle uptake, intracellular delivery of lytic cargo, or additional uncharacterized components inherited from NK92 cells such as endogenous surface proteins like natural cytotoxicity receptors (e.g., NKp30, NKp44, NKp46), NKG2D and DNAM‐1, which bind to stress‐induced ligands (like MICA/B, ULBPs) on cancer cells (Bottino et al. 2024), may explain the higher levels of cytotoxicity of untargeted NK92‐ACDVs than for T‐ACDVs. Importantly, as we did not quantify protein levels per vesicle, these data should be interpreted as showing that qualitative differences in death‐ligand repertoire do not directly translate into greater cytotoxicity, and that NK92‐ACDVs may provide a more efficient platform for delivering cytotoxic signals despite a narrower set of detected ligands.

NK92 cells are clinically safe and efficacious in patients with cancer (Tang et al. 2018; Williams et al. 2017), easier and cheaper to produce at clinical scale than activated‐T cells. Based on these properties of NK92 cells, we chose to continue the in vivo and ex vivo studies with NK92‐ACDVs.

In the SK‐OV‐3 xenograft NSG mouse model, the targeted NK92‐ACDV‐Nimotuzumab, reduced tumour volume relative to non‐targeted NK92‐ACDVs. This observation is different from the in vitro assay with SK‐OV‐3, where both targeted and untargeted NK92‐ACDVs showed similar cytotoxicity, highlighting the importance of targeting in mouse xenograft models.

In the ex vivo cytotoxicity with CLL patient cell biopsies, the targeted NK92‐ACDV‐14f7hT showed higher cytotoxicity relative to untargeted NK92‐ACDVs. These findings highlight the potential of targeted NK92‐ACDVs as a viable platform for cancer therapy and warrant further investigation for potential clinical applications.

In conclusion, while immune cell‐derived exosomes have shown significant potential in therapeutic applications, there are limitations associated with their production yield, heterogeneity, and targeting efficiency. ACDVs conjugated with antibodies offer a more controlled and efficient approach, addressing these limitations. The ability to produce ACDVs in larger quantities with specific targeting and enhanced penetration capabilities, makes them a promising alternative to natural exosomes for therapeutic delivery. Further research and development in this direction could lead to the creation of more effective therapeutic interventions with minimized off‐target effects.

## Author Contributions

**Brijesh Parlekar**: methodology, data curation, formal analysis, writing – original draft. **David W. Livingstone**: methodology, formal analysis. **Ashley R. Sutherland**: methodology, formal analysis. **Andrés X. Medina**: methodology, formal analysis. **Wendy Bernhard**: methodology. **John Decoteau**: investigation, resources. **Tays Hernández**: resources, writing – review and editing. **C. Ronald Geyer**: conceptualization, writing – original draft, investigation, supervision, project administration, writing – review and editing, funding acquisition.

## Funding

This study was supported by the Natural Sciences and Engineering Research Council of Canada, 25634.

## Ethics Statement

Ethics approval was obtained by the Biomedical Research Ethics Board at the University of Saskatchewan for this project, approval number 4369.

## Consent

The authors have nothing to report.

## Conflicts of Interest

The authors declare that they have no conflict of interest.

## Geolocation Information

This project was performed in Canada.

## Data Availability

The data that support the findings of this study are available from the corresponding author upon reasonable request.

## References

[jev270231-bib-0001] Afshar‐Sterle, S. , D. Zotos , N. J. Bernard , et al. 2014. “Fas Ligand–Mediated Immune Surveillance by T Cells Is Essential for the Control of Spontaneous B Cell Lymphomas.” Nature Medicine 20, no. 3: 283–290. 10.1038/nm.3442 24487434

[jev270231-bib-0002] Anel, A. , A. K. Simon , N. Auphan , et al. 1995. “Two Signaling Pathways Can Lead to Fas Ligand Expression in CD8+ Cytotoxic T Lymphocyte Clones.” European Journal of Immunology 25, no. 12: 3381–3387. 8566027 10.1002/eji.1830251227

[jev270231-bib-0003] Bellavia, D. , S. Raimondo , G. Calabrese , et al. 2017. “Interleukin 3‐Receptor Targeted Exosomes Inhibit In Vitro and In Vivo Chronic Myelogenous Leukemia Cell Growth.” Theranostics 7, no. 5: 1333–1345. 28435469 10.7150/thno.17092 PMC5399597

[jev270231-bib-0004] Bottino, C. , V. Picant , E. Vivier , and R. Castriconi . 2024. “Natural Killer Cells and Engagers: Powerful Weapons Against Cancer.” Immunological Reviews 328, no. 1: 412–421. 39180430 10.1111/imr.13384 PMC11659922

[jev270231-bib-0005] Castillo‐Peña, A. , and S. Molina‐Pinelo . 2023. “Landscape of Tumor and Immune System Cells‐Derived Exosomes in Lung Cancer: Mediators of Antitumor Immunity Regulation.” Frontiers in Immunology 14: 1279495. https://www.frontiersin.org/journals/immunology/articles/10.3389/fimmu.2023.1279495/full. 37915578 10.3389/fimmu.2023.1279495 PMC10616833

[jev270231-bib-0006] Choo, Y. W. , M. Kang , H. Y. Kim , et al. 2018. “M1 Macrophage‐Derived Nanovesicles Potentiate the Anticancer Efficacy of Immune Checkpoint Inhibitors.” ACS Nano 12, no. 9: 8977–8993. 30133260 10.1021/acsnano.8b02446

[jev270231-bib-0007] Crum, R. J. , H. Capella‐Monsonís , J. Chang , et al. 2023. “Biocompatibility and Biodistribution of Matrix‐Bound Nanovesicles In Vitro and In Vivo.” Acta Biomaterialia 155: 113–122. 36423817 10.1016/j.actbio.2022.11.026

[jev270231-bib-0008] Devaraj, N. K. , and M. G Finn . 2021. “Introduction: Click Chemistry.” Chemical Reviews 121, no. 12: 6697–6698. 34157843 10.1021/acs.chemrev.1c00469

[jev270231-bib-0009] Dorothée, G. , I. Vergnon , J. Menez , et al. 2002. “Tumor‐Infiltrating CD4+ T Lymphocytes Express APO2 Ligand (APO2L)/TRAIL Upon Specific Stimulation With Autologous Lung Carcinoma Cells: Role of IFN‐α on APO2L/TRAIL Expression and ‐Mediated Cytotoxicity1.” Journal of Immunology 169, no. 2: 809–817. 10.4049/jimmunol.169.2.809 12097384

[jev270231-bib-0010] Federici, C. , E. Shahaj , S. Cecchetti , et al. 2020. “Natural‐Killer‐Derived Extracellular Vesicles: Immune Sensors and Interactors.” Frontiers in Immunology 11: 262. https://www.frontiersin.org/journals/immunology/articles/10.3389/fimmu.2020.00262/full. 32231660 10.3389/fimmu.2020.00262 PMC7082405

[jev270231-bib-0011] Fernández‐Marrero, Y. , L. Roque‐Navarro , T. Hernández , et al. 2011. “A Cytotoxic Humanized Anti‐Ganglioside Antibody Produced in a Murine Cell Line Defective of N‐Glycolylated‐Glycoconjugates.” Immunobiology 216, no. 12: 1239–1247. 21802167 10.1016/j.imbio.2011.07.004

[jev270231-bib-0012] Fu, W. , C. Lei , S. Liu , et al. 2019. “CAR Exosomes Derived From Effector CAR‐T Cells Have Potent Antitumour Effects and Low Toxicity.” Nature Communications 10, no. 1: 4355. 10.1038/s41467-019-12321-3 PMC6761190 31554797

[jev270231-bib-0013] Gagnoux‐Palacios, L. , H. Awina , S. Audebert , et al. 2018. “Cell Polarity and Adherens Junction Formation Inhibit Epithelial Fas Cell Death Receptor Signaling.” Journal of Cell Biology 217, no. 11: 3839–3852. 30242034 10.1083/jcb.201805071 PMC6219722

[jev270231-bib-0014] Gao, J. , A. Li , J. Hu , L. Feng , L. Liu , and Z. Shen . 2023. “Recent Developments in Isolating Methods for Exosomes.” Frontiers in Bioengineering and Biotechnology 10: 1100892. 36714629 10.3389/fbioe.2022.1100892 PMC9879965

[jev270231-bib-0015] He, J. , W. Ren , W. Wang , et al. 2022. “Exosomal Targeting and Its Potential Clinical Application.” Drug Delivery and Translational Research 12, no. 10: 2385–2402. 34973131 10.1007/s13346-021-01087-1 PMC9458566

[jev270231-bib-0016] Hong, J. , M. Kang , M. Jung , et al. 2021. “T‐Cell‐Derived Nanovesicles for Cancer Immunotherapy.” Advanced Materials 33, no. 33: e2101110. 34235790 10.1002/adma.202101110

[jev270231-bib-0017] Jang, H. J. , K. S. Shim , J. Lee , et al. 2023. “Engineering of Cell Derived‐Nanovesicle as an Alternative to Exosome Therapy.” Tissue Engineering and Regenerative Medicine 21, no. 1: 1–19. 38066355 10.1007/s13770-023-00610-4 PMC10764700

[jev270231-bib-0018] Jong, A. Y. , C. H. Wu , J. Li , et al. 2017. “Large‐Scale Isolation and Cytotoxicity of Extracellular Vesicles Derived From Activated Human Natural Killer Cells.” Journal of Extracellular Vesicles 6, no. 1: 1294368. 28326171 10.1080/20013078.2017.1294368 PMC5345580

[jev270231-bib-0019] Jung, D. , S. Shin , S. M. Kang , et al. 2022. “Reprogramming of T Cell‐Derived Small Extracellular Vesicles Using IL2 Surface Engineering Induces Potent Anti‐Cancer Effects Through miRNA Delivery.” Journal of Extracellular Vesicles 11, no. 12: 12287. 36447429 10.1002/jev2.12287 PMC9709340

[jev270231-bib-0020] Kalluri, R. 2016. “The Biology and Function of Exosomes in Cancer.” Journal of Clinical Investigation 126, no. 4: 1208–1215. 27035812 10.1172/JCI81135 PMC4811149

[jev270231-bib-0021] Kim, H. Y. , H. K. Min , H. W. Song , et al. 2022. “Delivery of human Natural Killer Cell‐Derived Exosomes for Liver Cancer Therapy: An In Vivo Study in Subcutaneous and Orthotopic Animal Models.” Drug Delivery 29, no. 1: 2897–2911. 36068970 10.1080/10717544.2022.2118898 PMC9467548

[jev270231-bib-0022] Knight, M. J. , C. D. Riffkin , A. M. Muscat , D. M. Ashley , and C. J Hawkins . 2001. “Analysis of FasL and TRAIL Induced Apoptosis Pathways in Glioma Cells.” Oncogene 20, no. 41: 5789–5798. 11593384 10.1038/sj.onc.1204810

[jev270231-bib-0023] Kreuwel, H. T. C. , D. J. Morgan , T. Krahl , A. Ko , N. Sarvetnick , and L. A Sherman . 1999. “Comparing the Relative Role of Perforin/Granzyme Versus Fas/Fas Ligand Cytotoxic Pathways in CD8+ T Cell‐Mediated Insulin‐Dependent Diabetes Mellitus1.” Journal of Immunology 163, no. 8: 4335–4341. 10510373

[jev270231-bib-0024] Liang, Y. , L. Duan , J. Lu , and J. Xia . 2021. “Engineering Exosomes for Targeted Drug Delivery.” Theranostics 11, no. 7: 3183–3195. 33537081 10.7150/thno.52570 PMC7847680

[jev270231-bib-0025] Lieberman, J. 2003. “The ABCs of Granule‐Mediated Cytotoxicity: New Weapons in the Arsenal.” Nature Reviews Immunology 3, no. 5: 361–370. 10.1038/nri1083 12766758

[jev270231-bib-0026] Liu, C. G. , Y. Wang , P. Liu , et al. 2020. “Aptamer‐T Cell Targeted Therapy for Tumor Treatment Using Sugar Metabolism and Click Chemistry.” Acs Chemical Biology 15, no. 6: 1554–1565. 32401486 10.1021/acschembio.0c00164

[jev270231-bib-0027] Lopez, J. A. , O. Susanto , M. R. Jenkins , et al. 2013. “Perforin Forms Transient Pores on the Target Cell Plasma Membrane to Facilitate Rapid Access of Granzymes During Killer Cell Attack.” Blood 121, no. 14: 2659–2668. 23377437 10.1182/blood-2012-07-446146

[jev270231-bib-0028] Lugini, L. , S. Cecchetti , V. Huber , et al. 2012. “Immune Surveillance Properties of Human NK Cell‐Derived Exosomes.” Journal of Immunology 189, no. 6: 2833–2842. 10.4049/jimmunol.1101988 22904309

[jev270231-bib-0029] Malykh, Y. N. , R. Schauer , and L. Shaw . 2001. “N‐Glycolylneuraminic Acid in Human Tumours**.” Biochimie 83, no. 7: 623–634. 11522391 10.1016/s0300-9084(01)01303-7

[jev270231-bib-0030] Mazorra, Z. , L. Chao , A. Lavastida , et al. 2018. “Nimotuzumab: Beyond the EGFR Signaling Cascade Inhibition.” Seminars in Oncology 45, no. 1: 18–26. 30318080 10.1053/j.seminoncol.2018.04.008

[jev270231-bib-0031] Mirandola, P. , C. Ponti , G. Gobbi , et al. 2004. “Activated Human NK and CD8+ T Cells Express Both TNF‐related Apoptosis‐Inducing Ligand (TRAIL) and TRAIL Receptors But Are Resistant to TRAIL‐Mediated Cytotoxicity.” Blood 104, no. 8: 2418–2424. 15205263 10.1182/blood-2004-04-1294

[jev270231-bib-0032] Morad, G. , C. V. Carman , E. J. Hagedorn , et al. 2019. “Tumor‐Derived Extracellular Vesicles Breach the Intact Blood–Brain Barrier via Transcytosis.” ACS Nano 13, no. 12: 13853–13865. 31479239 10.1021/acsnano.9b04397 PMC7169949

[jev270231-bib-0033] Nakajima, H. , and P. A. Henkart . 1994. “Cytotoxic Lymphocyte Granzymes Trigger a Target Cell Internal Disintegration Pathway Leading to Cytolysis and DNA Breakdown.” Journal of Immunology 152, no. 3: 1057–1063. 7507956

[jev270231-bib-0034] Ng, C. Y. , L. T. Kee , M. E. Al‐Masawa , et al. 2022. “Scalable Production of Extracellular Vesicles and Its Therapeutic Values: A Review.” International Journal of Molecular Sciences 23, no. 14: 7986. 35887332 10.3390/ijms23147986 PMC9315612

[jev270231-bib-0035] Oh, K. , S. R. Kim , D. K. Kim , et al. 2015. “In Vivo Differentiation of Therapeutic Insulin‐Producing Cells From Bone Marrow Cells via Extracellular Vesicle‐Mimetic Nanovesicles.” ACS Nano 9, no. 12: 11718–11727. 26513554 10.1021/acsnano.5b02997

[jev270231-bib-0036] Ohno, S. , M. Takanashi , K. Sudo , et al. 2013. “Systemically Injected Exosomes Targeted to EGFR Deliver Antitumor MicroRNA to Breast Cancer Cells.” Molecular Therapy 21, no. 1: 185–191. 23032975 10.1038/mt.2012.180 PMC3538304

[jev270231-bib-0037] Oshimi, Y. , S. Oda , Y. Honda , S. Nagata , and S. Miyazaki . 1996. “Involvement of Fas Ligand and Fas‐mediated Pathway in the Cytotoxicity of human Natural Killer Cells.” Journal of Immunology 157, no. 7: 2909–2915. 8816396

[jev270231-bib-0038] Ou, Y. H. , J. Liang , W. H. Chng , et al. 2022. “Investigations on Cellular Uptake Mechanisms and Immunogenicity Profile of Novel Bio‐Hybrid Nanovesicles.” Pharmaceutics 14, no. 8: 1738. 36015364 10.3390/pharmaceutics14081738 PMC9413569

[jev270231-bib-0039] Pardo, J. , S. Balkow , A. Anel , and M. M Simon . 2002. “Granzymes Are Essential for Natural Killer Cell‐mediated and Perf‐facilitated Tumor Control.” European Journal of Immunology 32, no. 10: 2881–2886. 12355441 10.1002/1521-4141(2002010)32:10<2881::AID-IMMU2881>3.0.CO;2-K

[jev270231-bib-0040] Ramakrishnan, M. S. , A. Eswaraiah , T. Crombet , et al. 2009. “Nimotuzumab, a Promising Therapeutic Monoclonal for Treatment of Tumors of Epithelial Origin.” mAbs 1, no. 1: 41–48. 20046573 10.4161/mabs.1.1.7509 PMC2715181

[jev270231-bib-0041] Rao, L. , L. Wu , Z. Liu , et al. 2020. “Hybrid Cellular Membrane Nanovesicles Amplify Macrophage Immune Responses Against Cancer Recurrence and Metastasis.” Nature Communications 11, no. 1: 4909. 10.1038/s41467-020-18626-y PMC7527506 32999291

[jev270231-bib-0042] Ruan, H. , Y. Li , C. Wang , et al. 2023. “Click Chemistry Extracellular Vesicle/Peptide/Chemokine Nanocarriers for Treating Central Nervous System Injuries.” Acta Pharmaceutica Sinica B 13, no. 5: 2202–2218. 37250158 10.1016/j.apsb.2022.06.007 PMC10213615

[jev270231-bib-0043] Ruan, Q. , and C. Zhao . 2023. “A Method for Parallel Microscale Protein Labeling and Precise Control Over the Average Degree of Labeling (aDoL).” Scientific Reports 13, no. 1: 8961. 37268718 10.1038/s41598-023-36163-8 PMC10238424

[jev270231-bib-0044] Samraj, A. N. , H. Läubli , N. Varki , and A. Varki . 2014. “Involvement of a Non‐Human Sialic Acid in Human Cancer.” Frontiers in oncology 4: 33. 24600589 10.3389/fonc.2014.00033 PMC3928833

[jev270231-bib-0045] Seo, N. , Y. Shirakura , Y. Tahara , et al. 2018. “Activated CD8+ T Cell Extracellular Vesicles Prevent Tumour Progression by Targeting of Lesional Mesenchymal Cells.” Nature Communications 9, no. 1: 1–11. 10.1038/s41467-018-02865-1 PMC5789986 29382847

[jev270231-bib-0046] Shin, S. , I. Jung , D. Jung , et al. 2022. “Novel Antitumor Therapeutic Strategy Using CD4+ T Cell‐Derived Extracellular Vesicles.” Biomaterials 289: 121765. 36067566 10.1016/j.biomaterials.2022.121765

[jev270231-bib-0047] Smyth, T. , K. Petrova , N. M. Payton , et al. 2014. “Surface Functionalization of Exosomes Using Click Chemistry.” Bioconjugate Chemistry 25, no. 10: 1777–1784. 25220352 10.1021/bc500291r PMC4198107

[jev270231-bib-0048] Straus, S. E. , E. S. Jaffe , J. M. Puck , et al. 2001. “The Development of Lymphomas in Families With Autoimmune Lymphoproliferative Syndrome With Germline Fas Mutations and Defective Lymphocyte Apoptosis.” Blood 98, no. 1: 194–200. 11418480 10.1182/blood.v98.1.194

[jev270231-bib-0049] Weltgesundheitsorganisation . 2017. “WHO Classification of Tumours of Haematopoietic and Lymphoid tissues.” 4th ed. In Lyon: International Agency for Research on Cancer, edited by Swerdlow, SH , Campo, E , Harris, NL , et al., 585. World Health Organization Classification of Tumours.

[jev270231-bib-0050] Takayama, Y. , K. Kusamori , and M. Nishikawa . 2019. “Click Chemistry as a Tool for Cell Engineering and Drug Delivery.” Molecules (Basel, Switzerland) 24, no. 1: 172. 30621193 10.3390/molecules24010172 PMC6337375

[jev270231-bib-0051] Takeda, K. , Y. Hayakawa , M. J. Smyth , et al. 2001. “Involvement of Tumor Necrosis Factor‐related Apoptosis‐Inducing Ligand in Surveillance of Tumor Metastasis by Liver Natural Killer Cells.” Nature Medicine 7, no. 1: 94–100. 10.1038/83416 11135622

[jev270231-bib-0052] Takeda, K. , M. J. Smyth , E. Cretney , et al. 2002. “Critical Role for Tumor Necrosis Factor–Related Apoptosis‐Inducing Ligand in Immune Surveillance Against Tumor Development.” Journal of Experimental Medicine 195, no. 2: 161–169. 11805143 10.1084/jem.20011171 PMC2193611

[jev270231-bib-0053] Tang, X. , L. Yang , Z. Li , et al. 2018. “First‐in‐man Clinical Trial of CAR NK‐92 Cells: Safety Test of CD33‐CAR NK‐92 Cells in Patients With Relapsed and Refractory Acute Myeloid Leukemia.” American Journal of Cancer Research 8, no. 6: 1083–1089. 30034945 PMC6048396

[jev270231-bib-0054] Tao, B. , R. Du , X. Zhang , et al. 2023. “Engineering CAR‐NK Cell Derived Exosome Disguised Nano‐Bombs for Enhanced HER2 Positive Breast Cancer Brain Metastasis Therapy.” Journal of Controlled Release 363: 692–706. 37813124 10.1016/j.jconrel.2023.10.007

[jev270231-bib-0055] Tian, T. , H. X. Zhang , C. P. He , et al. 2018. “Surface Functionalized Exosomes as Targeted Drug Delivery Vehicles for Cerebral Ischemia Therapy.” Biomaterials 150: 137–149. 29040874 10.1016/j.biomaterials.2017.10.012

[jev270231-bib-0056] Van Deun, J. , Q. Roux , S. Deville , et al. 2020. “Feasibility of Mechanical Extrusion to Coat Nanoparticles With Extracellular Vesicle Membranes.” Cells 9, no. 8: 1797. 32751082 10.3390/cells9081797 PMC7464356

[jev270231-bib-0057] Wang, W. , Z. Zhao , Z. Zhang , et al. 2017. “Redirecting Killer T Cells Through Incorporation of Azido Sugars for Tethering Ligands.” ChemBioChem: A European Journal of Chemical Biology 18, no. 21: 2082–2086. 28862366 10.1002/cbic.201700340

[jev270231-bib-0058] Williams, B. A. , A. D. Law , B. Routy , et al. 2017. “A Phase I Trial of NK‐92 Cells for Refractory Hematological Malignancies Relapsing After Autologous Hematopoietic Cell Transplantation Shows Safety and Evidence of Efficacy.” Oncotarget 8, no. 51: 89256–89268. 29179517 10.18632/oncotarget.19204 PMC5687687

[jev270231-bib-0059] Wu, C. H. , J. Li , L. Li , et al. 2019. “Extracellular Vesicles Derived From Natural Killer Cells Use Multiple Cytotoxic Proteins and Killing Mechanisms to Target Cancer Cells.” Journal of Extracellular Vesicles 8, no. 1: 1588538. 30891164 10.1080/20013078.2019.1588538 PMC6419691

[jev270231-bib-0060] Yu, Y. , Q. Cheng , X. Ji , et al. 2022. “Engineered Drug‐loaded Cellular Membrane Nanovesicles for Efficient Treatment of Postsurgical Cancer Recurrence and Metastasis.” Science Advances 8, no. 49: eadd3599. 36490349 10.1126/sciadv.add3599 PMC9733928

[jev270231-bib-0061] Zhu, L. , P. Gangadaran , S. Kalimuthu , et al. 2018. “Novel Alternatives to Extracellular Vesicle‐based Immunotherapy—exosome Mimetics Derived From Natural Killer Cells.” Artificial Cells, Nanomedicine, and Biotechnology 46, no. S3: S166–S179. 30092165 10.1080/21691401.2018.1489824

